# Cardioprotection by isosteviol derivate JC105: A unique drug property to activate ERK1/2 only when cells are exposed to hypoxia‐reoxygenation

**DOI:** 10.1111/jcmm.15721

**Published:** 2020-08-14

**Authors:** Khaja Shameem Mohammed Abdul, Jayachandra Rayadurgam, Neha Faiz, Aleksandar Jovanović, Wen Tan

**Affiliations:** ^1^ Institute of Biomedical and Pharmaceutical Sciences Guangdong University of Technology Guangzhou PR China; ^2^ Research Center for Drug Discovery School of Pharmaceutical Sciences Sun Yat‐Sen University Guangzhou PR China; ^3^ Department of Basic and Clinical Sciences University of Nicosia Medical School Nicosia Cyprus; ^4^ Centre for Neuroscience and Integrative Brain Research (CENIBRE) University of Nicosia Medical School Nicosia Cyprus; ^5^ Jeffrey Cheah School of Medicine and Health Sciences Monash University Malaysia Selangor Darul Ehsan Malaysia

**Keywords:** cardioprotection, ERK, H9c2 cells, hypoxia‐reoxygenation

## Abstract

In the present study, we have investigated potential cardioprotective properties of Isosteviol analogue we recently synthesized and named JC105. Treatment of heart embryonic H9c2 cells with JC105 (10 μM) significantly increased survival of cells exposed to hypoxia‐reoxygenation. JC105 (10 μM) activated ERK1/2, DRP1 and increased levels of cardioprotective SUR2A in hypoxia‐reoxygenation, but did not have any effects on ERK1/2, DRP1 and/or SUR2A in normoxia. U0126 (10 μM) inhibited JC105‐mediated phosphorylation of ERK1/2 and DRP1 without affecting AKT or AMPK, which were also not regulated by JC105. Seahorse bioenergetic analysis demonstrated that JC105 (10 μM) did not affect mitochondria at rest, but it counteracted all mitochondrial effects of hypoxia‐reoxygenation. Cytoprotection afforded by JC105 was inhibited by U0126 (10 μM). Taken all together, these demonstrate that (a) JC105 protects H9c2 cells against hypoxia‐reoxygenation and that (b) this effect is mediated via ERK1/2. The unique property of JC105 is that selectively activates ERK1/2 in cells exposed to stress, but not in cells under non‐stress conditions.

## 1 INTRODUCTION

Cardioprotection can be defined as a property of cardiac muscle to withstand challenges by virtue of intracellular signalling pathways that, when activated, increase cellular resistance to metabolic challenges including ischaemia‐reperfusion. It is a consensus view that development of clinically viable and safe therapeutic cardioprotective strategies is warranted. It is generally accepted that a cardioprotective‐based strategy would be useful in the therapy of ischaemic heart disease and other cardiac diseases. Such therapy would not be mutually exclusive with traditional therapies aimed to reconstitute coronary blood flow or decrease myocardial metabolic demands, but would be rather complementary to those therapies.^1^

Isosteviol (STV) is a glycan of stevioside, which has been widely used in Paraguay since 16th century, and is currently used as a food supplement in over 90 countries.^2^ It has been suggested that STV is a pharmacologically active substance deserving to be tested as a potential therapy for diabetes mellitus, hypertension and heart failure.^2^ We have recently synthesized two series of analogues from isosteviol with modifications at C‐16, C‐19 positions (in series one) and at C‐15, C‐16 positions (in series two). We have demonstrated that one of those analogues (compound 9), named JC105, protects zebrafish embryos against doxorubicin‐induced cardiotoxicity in vivo. This particular finding suggests that this compound could have cardioprotective properties.^3^

Therefore, we decided to test cardioprotection afforded by JC105. To do that, we have applied hypoxia‐reoxygenation challenge on rat heart embryonic H9c2 cells, which is an experimental model used to study cardioprotection in quick and reliable manner.^1^, ^4^

We have found that JC105 protects H9c2 against hypoxia‐reoxygenation by selectively activating ERK1/2 during hypoxia‐reoxygenation without affecting ERK1/2 signalling pathway under normoxic conditions. This is the first account ever of a compound having such properties.

## 2 MATERIALS AND METHODS

### 2.1 Materials

JC105 was synthesized in our laboratory (see below). Isosteviol was purchased from Sigma–Aldrich Co. Ltd. U0126 was purchased from Cell Signaling Technology. 3‐[4, 5‐dimethylthiazol‐2‐yl] 2, 5‐diphenyl‐tetrazolium bromide (MTT) was purchased from VWR Life Sciences. Dulbecco's modified Eagle's medium (DMEM), foetal bovine serum (FBS), 0.05% Trypsin‐EDTA (1×), penicillin (10 000 U/mL)‐streptomycin (10 000 µg/mL) (Pen Strep), phosphate‐buffered saline (PBS, 1×), no glucose and no sodium pyruvate DMEM were purchased from Gibco. RIPA buffer was obtained from Biosharp. Protease inhibitor (100x) was obtained from Beyotime. Bicinochoninic acid (BCA) assay kit was obtained from Beyotime. Stock concentration of JC105 (100 mM) and U0126 (10 mM) was dissolved in DMSO. MTT (5mg/ml) was dissolved in DMEM. During all treatments, the concentration of DMSO was below 0.1% (v/v). Phospho‐ERK 1/2 (Thr^202^/Tyr^204^), Total‐ERK1/2, Phospho‐DRP1 (Ser^616^), Total‐DRP1, Phospho‐AKT (Ser^473^) and Total‐AKT were obtained from Cell Signaling Technology. Phospho‐AMPKα (Thr^172^) and Total‐AMPK were obtained from Abcam and GAPDH was obtained from Proteintech. Anti‐rabbit IgG and anti‐mouse IgG were obtained from Proteintech. Enhanced chemiluminescence reagent was obtained from Affinity Biosciences.

### 2.2 JC105

JC105 (4R,4aS,7S,8R,9S,11aS,11bS)‐7‐formyl‐8‐hydroxy‐4,9,11b‐trimethyltetradecahydro‐6a,9‐methanocyclohepta[a]naphthalene‐4‐carboxylic acid) was synthesized from isosteviol (ent‐16‐ketobeyeran‐19‐oic acid) as described with details in our recent work (compound 9 in Ref. [3]). The effect of concentration of 10 μM of JC105 was selected to be studied based on our preliminary data (see also Ref. [3]).

### 2.3 H9c2 cells and treatment

The H9c2 cardiomyocytes were obtained from the Cell Bank of Chinese Academy of Science. Cells were cultured in tissue flask containing DMEM supplemented with 2 mM l‐glutamine, 110 mg/L sodium pyruvate, 10% (v/v) FBS, 1% (v/v) Pen Strep and incubated at 37°C in humidified environment (5% CO_2_ and 95% air). The cells were seeded in 6 well plates and maintained until they reach 80%‐90% confluence. For some experiments, JC105 (10 μM) was treated only for 24 hours. For some experiments, the cells were pre‐treated with JC105 (10 μM) for 24 hours and continued treatment during 24 hours 1% hypoxia (using no glucose and no sodium pyruvate DMEM) and 4 hours reoxygenation (using fully supplemented DMEM and normoxia). For some experiments, U0126 (10 μM) was pre‐treated for 1 hour and then continued treatment with or without JC105 and performed hypoxia‐reoxygenation.

### 2.4 MTT‐based cell viability assay

Viability of the cells was assessed using 3‐[4, 5‐dimethylthiazol‐2‐yl]2, 5‐diphenyl‐tetrazolium bromide (MTT) assay. Briefly, H9c2 cardiomyocytes were plated in a 96‐well plate at 15 × 10^4^ cells/well and cultured until they reach 80%‐90% confluence with fully supplemented DMEM. JC105 (10 μM) was either treated for only 24 hours or after 24 hours pre‐treatment cells were continued treatment during 24 hours 1% hypoxia and 4 hours reoxygenation. Similarly, U0126 (10 μM) was pre‐treated for 1 hour after with or without JC105 pre‐treatment and then continued treatment in the presence or absence of JC105 during hypoxia‐reoxygenation. At the end of the experiment time period, freshly prepared MTT solution (0.5 mg/mL) was added to each well and incubated for 2 hours at 37°C. Immediately after incubation, medium of each was replaced with 150 μL of DMSO and the absorbance was measured at 570 nm using a microplate reader (TriStar2 LB 942, Berthold Technologies).

### 2.5 Western blot analysis

For Western blotting, H9c2 cells were lysed using RIPA buffer containing protease inhibitors (1×) (Biosharp). The cell lysates were collected, vortexed and centrifuged at 4°C for 10 min at 13 000× *g* to remove cell debris. The supernatant was transferred into new tubes, snap frozen in liquid nitrogen and stored at −80°C until further use. Bicinochoninic acid (BCA) assay (Beyotime) was used to measure the protein concentrations in the samples. For each sample, 20‐40 µg of protein was used to perform SDS‐PAGE and transferred to PVDF membrane. For all blots, overnight incubation at 4°C was performed for antibodies against Phospho‐ERK 1/2 (Thr^202^/Tyr^204^), Total‐ERK1/2, Phospho‐DRP1 (Ser^616^), Total‐DRP1, Phospho‐AKT (Ser^473^) and Total‐AKT, Phospho‐AMPKα (Thr^172^), Total‐AMPK and GAPDH. 1:1000 dilution was used for all antibodies. All the blots were incubated in TBST with 5% skimmed milk, and either phospho or total protein was determined using horse‐radish peroxidase conjugated secondary antibodies (anti‐rabbit IgG or anti‐mouse IgG) and enhanced chemiluminescence reagent. The Western blot band intensities were analysed using Quantiscan software. All the Western blots were performed in triplicates.

### 2.6 Bioenergetic analysis

Oxygen Consumption Rate (OCR) and Extracellular Acidification Rate (ECAR) of H9c2 cells were measured using the XF Cell Mito Stress Test and XF Glycolysis Stress Test in real‐time using Seahorse XF96 extracellular flux analyser (Seahorse Bioscience). H9c2 cells (30 000 cells/well) were seeded on XF96 cell culture plate and incubated overnight at 37°C in humidified environment (5% CO_2_ and 95% air) and followed by hypoxia‐reoxygenation challenge as described in treatment protocol. At the end of reoxygenation, cell culture media were replaced by XF assay medium (180 µL) to each well and then pre‐incubated for 1 hour at 37°C in humidified incubator without CO_2_. A day prior to seahorse experiment, XF sensor cartridge was hydrated with sterile deionised water (200 µL) and incubated overnight at 37°C in a humidified atmosphere without CO_2_. The next day, deionised water was replaced with 200 µL of XF calibrant and incubated for 1 hour prior to the experiment at 37°C in humidified atmosphere without CO_2_. For OCR analysis, 1 mmol/L sodium pyruvate, 2 mmol/L l‐glutamine and 10 mmol/L glucose were added into the XF assay medium and pH was adjusted to 7.4. After measuring basal respiration, oligomycin (1 μmol/L), carbonyl cyanide (trifluoromethoxy) phenylhydrazone (FCCP) (0.5 μmol/L) and rotenone/antimycin A (R/A) (1 μmol/L) were injected in sequence from port A, B and C respectively. In the ECAR assay, 2 mmol/L l‐glutamine was added to XF assay medium and pH was adjusted to 7.4. Glycolytic flux (glycolytic reserve and glycolytic capacity) was recorded by sequence addition of glucose (10 mmol/L), oligomycin (1 μmol/L) and 2‐deoxyglucose (50 mmol/L) from Port A, B and C, respectively. The hydrated XF sensor cartridge plates were loaded on XF prep station incubator and pre‐incubated at 37°C for 20 minutes and then plates with cells were introduced and run on the XF96 analyser to record OCR and ECAR values. OCR and ECAR were automatically recorded during specific time periods (with three readings each) by the Seahorse XF‐96 software and obtained readings of OCR and ECAR were normalized for total protein/well.

### 2.7 Statistical analysis

Data were analysed with a one‐way analysis of variance (ANOVA) using SigmaPlot 12.5 (Jandel Scientific). All the results were expressed as means ± SEM and the ‘n’ represents number of independent experiments. *P* < .05 was considered statistically significant.

## 3 RESULTS

### 3.1 JC105 protects H9c2 cells against hypoxia‐reoxygenation

**FIGURE 1:**
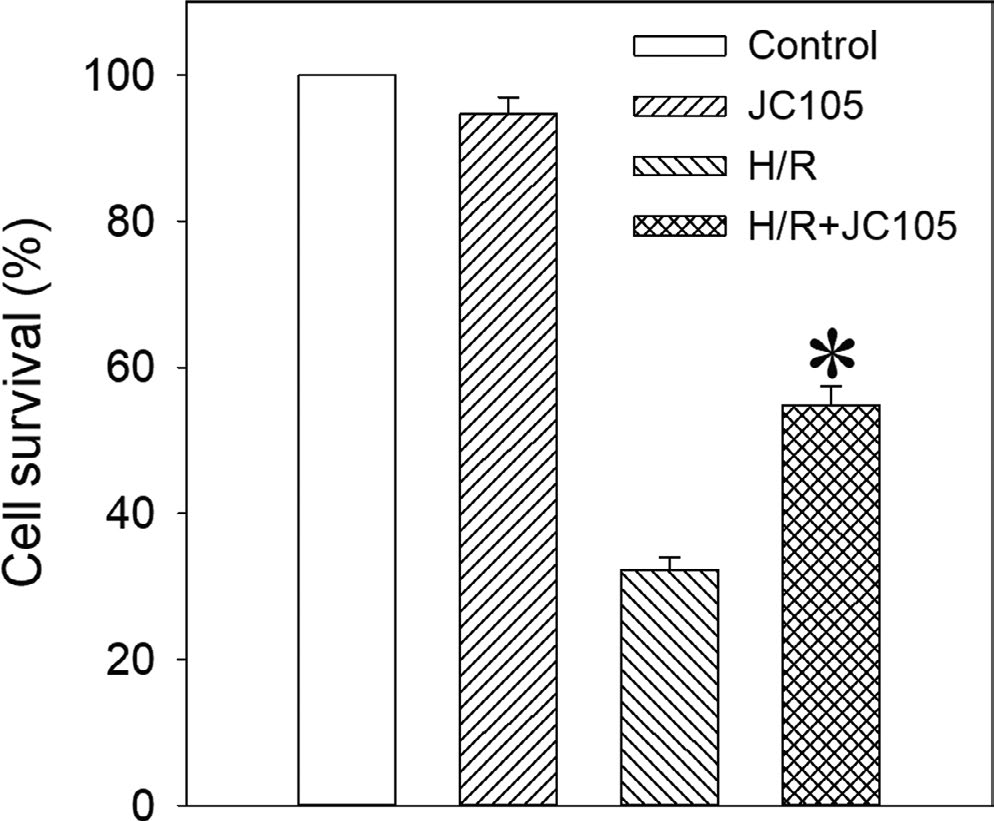
JC105 protects H9c2 cells against hypoxia‐reoxygenation. Bar graph depicting cell survival under normoxic conditions and under hypoxia‐reoxygenation (H/R) in the absence (control or H/R respectively) and presence of 10 μM JC105 (JC105 or H/R + JC105 respectively). Each bar represents mean ± SEM (n = 6‐10). *P < .001 when compared to hypoxia‐reoxygenation alone (H/R)

Exposure to JC105 (10 μM) under normoxic control condition did not significantly affect cellular viability (Figure 1), while exposure to hypoxia‐reoxygenation induced cell death (Figure 1). Treatment with JC105 (10 μM) significantly increased survival of cells exposed to hypoxia‐reoxygenation (from 32.2% ± 1.8% survived cells in the absence to 54.4% ± 2.7% survived cells in the presence of 10 μM JC105; *P* < .001; n = 6‐10 for each; Figure 1).

### 3.2 JC105 treatment alone under normoxic condition does not activate known cardioprotective signalling pathway

When H9c2 cells were treated with JC105 (10 μM) alone under normoxic condition for 24 hours, it did not affect the phosphorylation levels of ERK, AKT and AMPK. Furthermore, it also did not affect the levels of SUR2A and GAPDH (Figure S1).

### 3.3 JC105 moderately but significantly activates ERK1/2 during hypoxia‐reoxygenation

**FIGURE 2:**
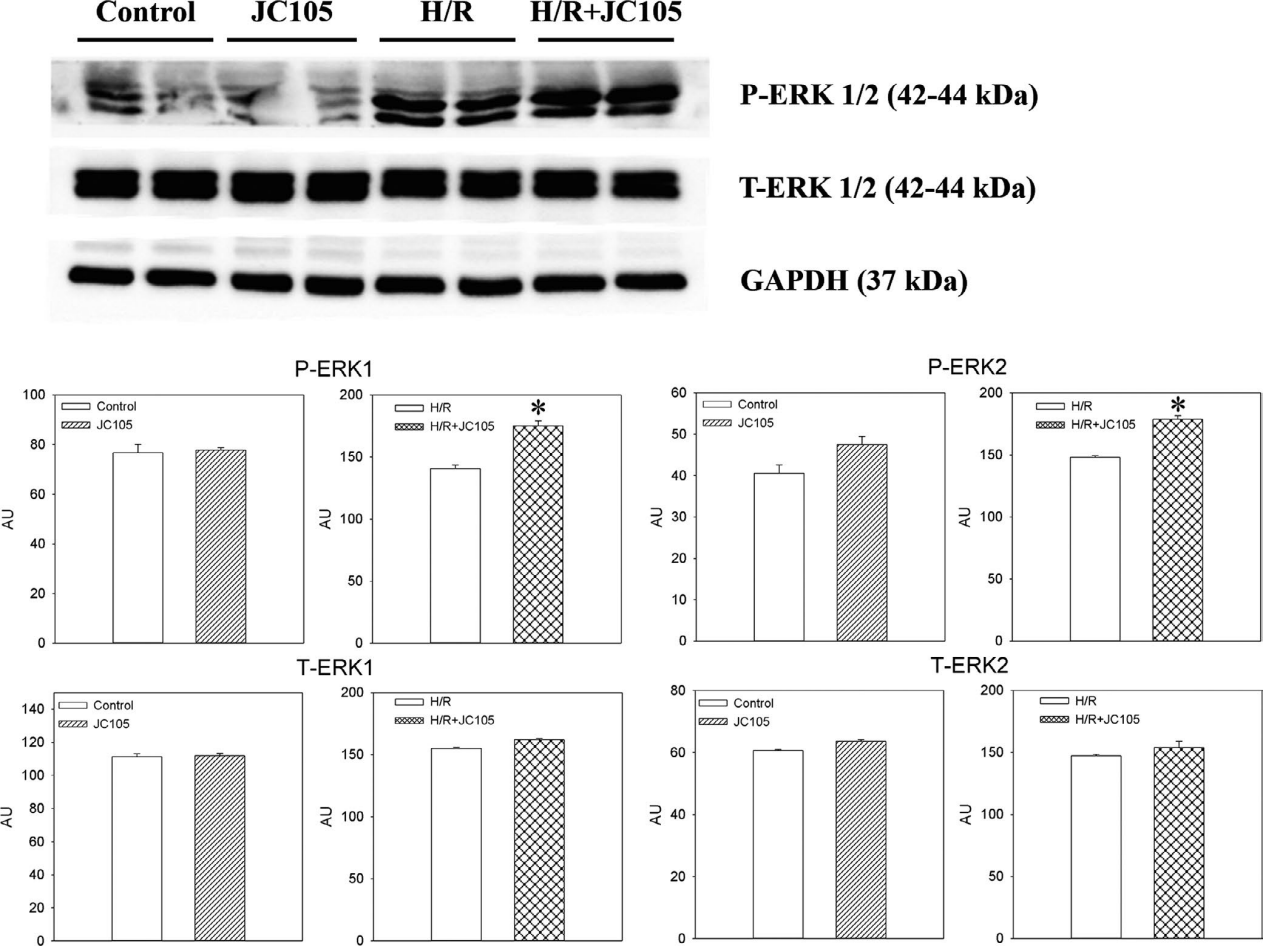
JC105 phosphorylates ERK1/2 in hypoxia‐reoxygenation while it does not affect ERK1/2 under normoxic conditions. Original Western blots and corresponding graphs for P‐ERK1/P‐ERK2 and T‐ERK1/T‐ERK2 under depicted conditions. Each bar represents mean ± SEM (n = 3‐4). *P < .001. Applied JC105 concentration was 10 μM

Treatment with JC105 (10 μM) did not affect levels of phospho‐ERK1 (P‐ERK1) and phospho‐ERK2 (P‐ERK2) nor total levels of ERK1 and ERK2 in H9c2 cells under normoxic conditions (Figure 2). However, when cells were treated with JC105 (10 μM) in hypoxia‐reoxygenation, phosphorylation of both ERK1 and ERK2 was moderately, but significantly, increased (PERK1: 140.6 ± 2.9AU without and 175.0 ± 4.1AU with JC105, *P* < .001; PERK2: 148.0 ± 1.4AU without and 178.8 ± 2.8AU with JC105, *P* < .001; Figure 2). Levels of total ERK1 and ERK2 were not affected by 10 μM JC105 either under control normoxic conditions or in hypoxia‐reoxygenation (Figure 2). ERK1/2 is established regulators of levels of cardioprotective SUR2A.^5^ JC105 (10 μM) did not affect SUR2A levels under normoxia (Figure S2). On the other hand, treatment of H9c2 cells with 10 μM JC105 significantly increased SUR2A levels and U0126 treatment reduced SUR2A levels (Figure S2).

### 3.4 U0126 inhibits JC105‐induced ERK1/2 and DRP1 phosphorylation in hypoxia‐reoxygenation without affecting phosphorylation of AKT and AMPKα

**FIGURE 3:**
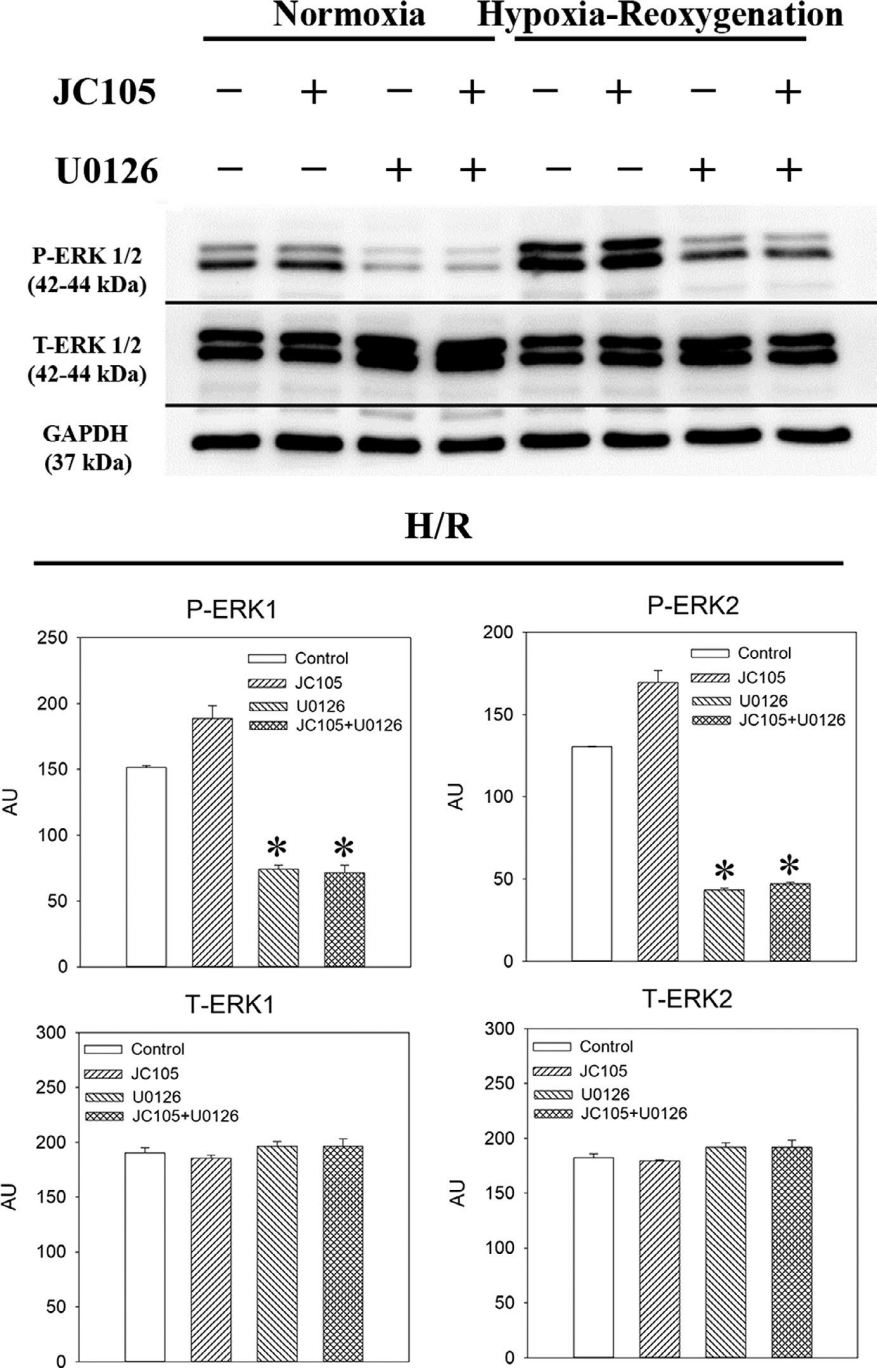
U0126 inhibits JC105‐induced ERK1/2 phosphorylation. Original Western blots and corresponding graphs for P‐ERK1/P‐ERK2 and T‐ERK1/T‐ERK2 under depicted conditions. *P < .001 when compared to the control. Each bar represents mean ± SEM (n = 3). Applied U0126 and JC105 concentrations were for 10 μM each

**FIGURE 4:**
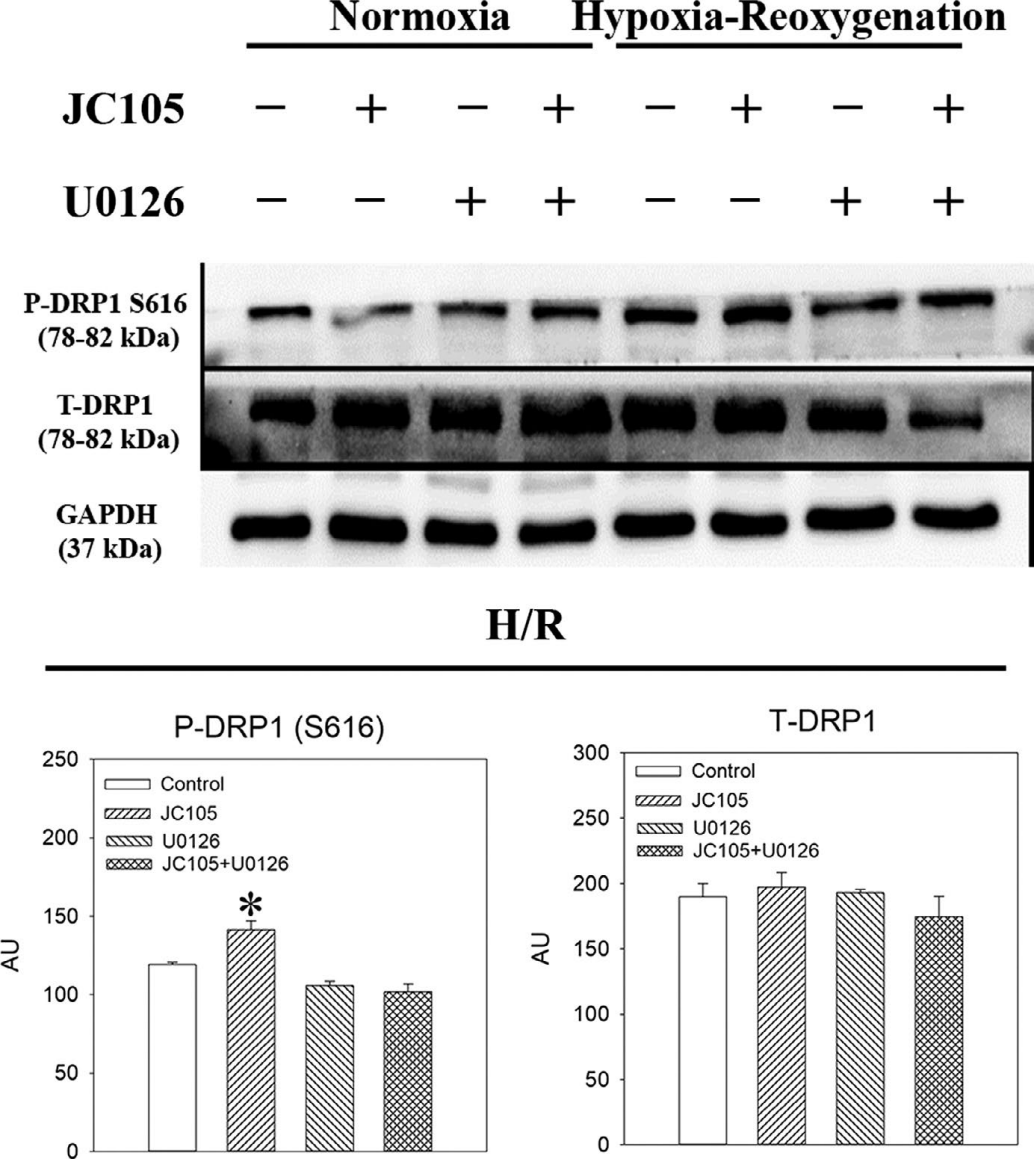
U0126 inhibits JC105‐induced DRP1 phosphorylation. Original Western blots and corresponding graphs for P‐DRP1 and T‐DRP1 under depicted conditions. *P < .001 when compared to the control. Each bar represent mean ± SEM (n = 3). Applied U0126 and JC105 concentrations were 10 μM for each

**FIGURE 5:**
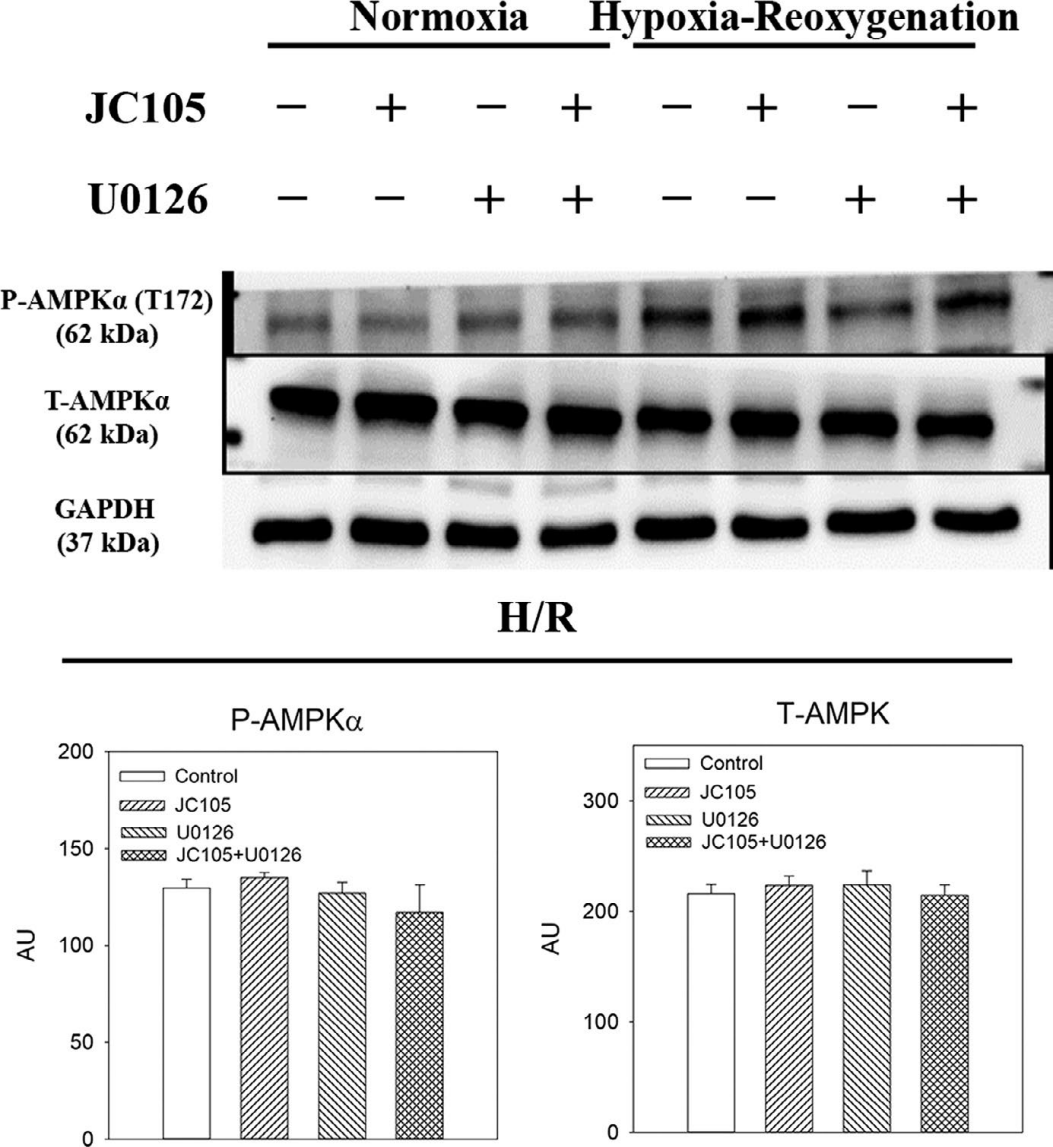
U0126 and JC105 do not affect AMPK. Original Western blots and corresponding graphs for P‐AMPKα and T‐AMPK under depicted conditions. Each bar represents mean ± SEM (n = 3). Applied U0126 and JC105 concentrations were 10 μM for each

**FIGURE 6:**
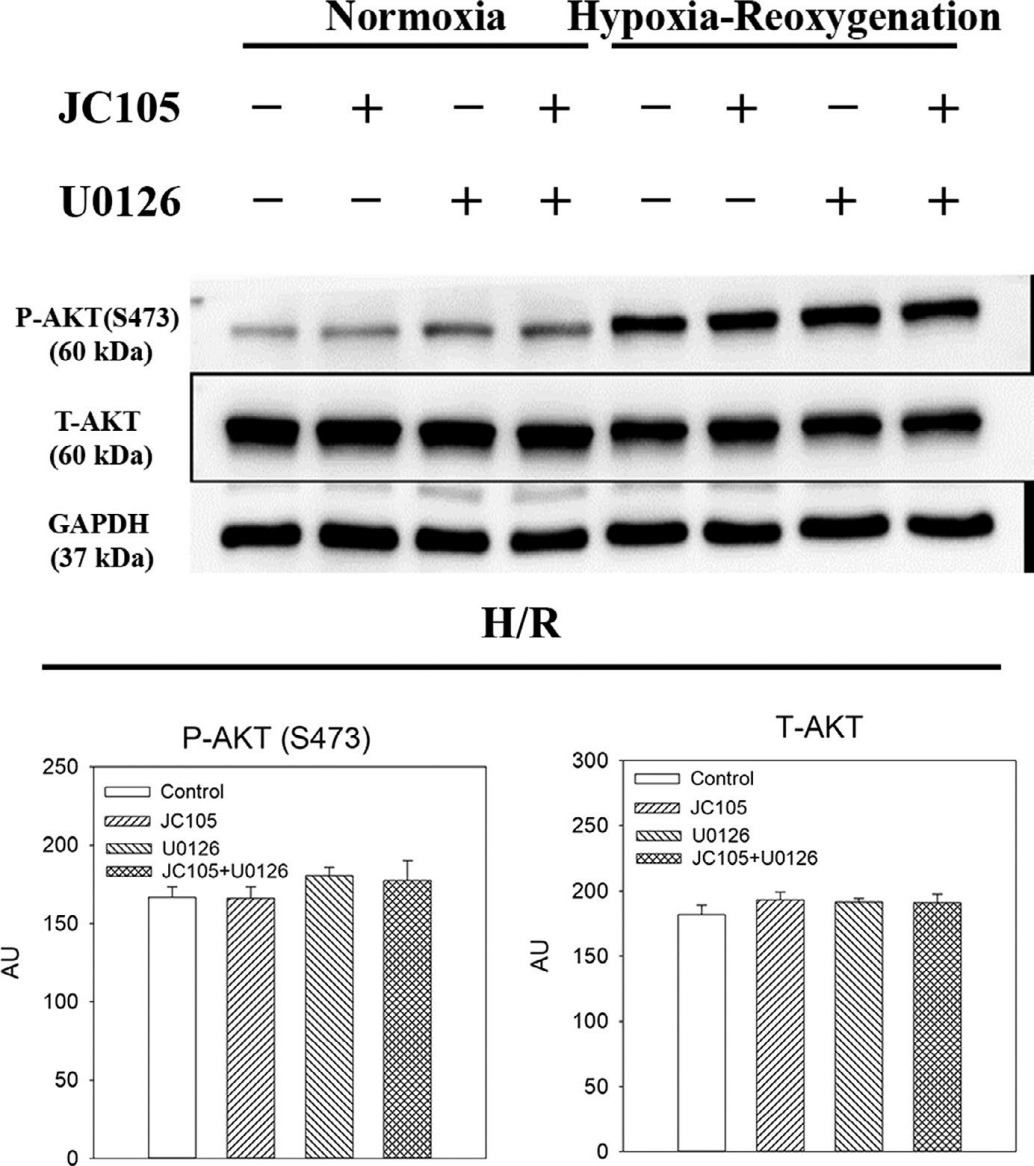
U0126 and JC105 do not affect AKT. Original Western blots and corresponding graphs for P‐AKT and T‐AKT under depicted conditions. Each bar represents mean ± SEM (n = 3). Applied U0126 and JC105 concentrations were 10 μM for each

To test a causal relationship between ERK1/2 phosphorylation and cytoprotection, we have examined the effect of U0126, a known MEK inhibitor. Treatment with U0126 alone (10 μM) significantly decreased levels of P‐ERK1 and P‐ERK2 (Figure 3) without affecting total levels of ERK1 and ERK2 (Figure 3). Concomitant treatment with U0126 (10 μM) and JC105 (10 μM) produced similar effects as U0126 (10 μM) alone (Figure 3), that is U0126 (10 μM) blocked JC105‐induced phosphorylation of ERK1 and ERK2. It has been recently shown that ERK1/2 targets DRP1 to regulate mitochondrial fission which is an essential process in mitochondrial and cellular adaptation to metabolic stress.^6^ JC105 (10 μM) moderately, but significantly, increased level of phospho‐DRP1 (P‐DRP1) in H9c2 cells exposed to hypoxia‐reoxygenation without effect on total DRP1 (Figure 4). This effect of JC105 was blocked by U0126 (Figure 4). JC105 (10 μM) nor U0126 (10 μM) did not have any effects on phosphorylation of AKT or AMPKα or total levels of AKT or AMPK (Figures 5 and 6).

### 3.5 JC105 improved (and U0126 inhibited JC105 induced recovery) mitochondrial function and glycolytic capacity of H9c2 cells in H/R injury

**FIGURE 7:**
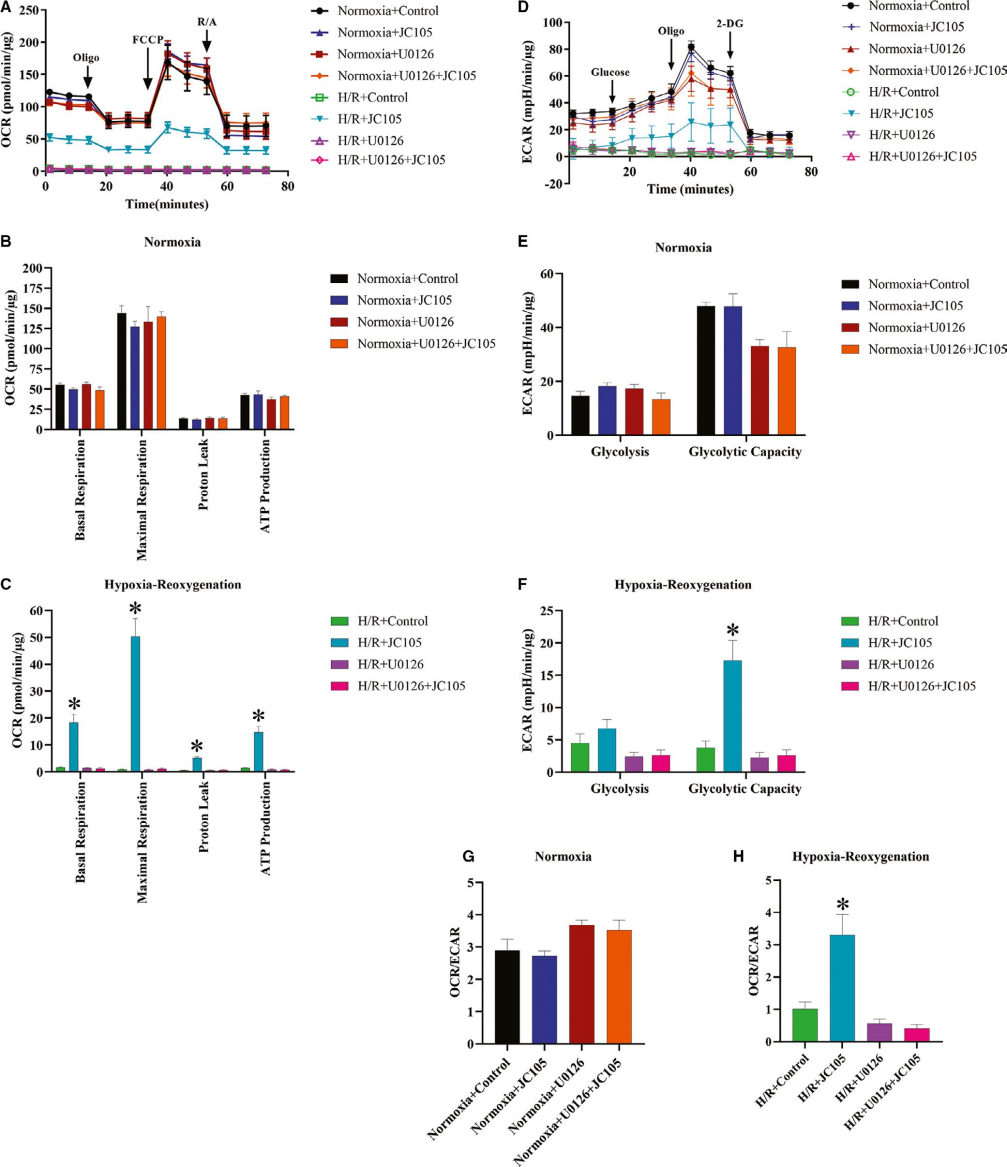
JC105 improved mitochondrial respiration and increased glycolytic capacity in hypoxia‐reoxygenation injury, whereas U0126 inhibited JC105‐mediated effects. A, Mitochondrial stress test was performed by sequential addition of 1 µmol/L oligomycin, 0.5 µmol/L FCCP and 0.5 µmol/L rotenone/antimycin A. B and C, Basal and maximal respiration, proton leak and ATP production (from left to right) under normoxia and hypoxia‐reoxygenation conditions are shown. D, Glycolysis stress test was performed by sequential addition of 10 mmol/L glucose, 1 µmol/L oligomycin and 50 mmol/L 2‐DG. E and F, Glycolysis and glycolytic capacity under normoxia and hypoxia‐reoxygenation conditions are shown. G and H, The ratio of OCR/ECAR under normoxia and hypoxia‐reoxygenation conditions. Oligo: oligomycin, an ATP synthase blocker; FCCP: carbonyl cyanide 4‐(trifluoromethoxy) phenylhydrazone; R/A: rotenone and antimycin A; 2‐DG: 2‐deoxyglucose. All data are shown as the mean ± SEM. *P < .001 when compared to the control

To test the effects of JC105 on cellular bioenergetics, we assessed mitochondrial respiration and glycolytic activity of H9c2 cells by measuring using OCR and ECAR under normoxia and H/R (Figure 7A,D). Under normoxic conditions, we found that OCR (Figure 7B) and ECAR (Figure 7E) were not significantly different among treatment groups. However, compared with the control cells, H/R significantly decreased mitochondrial respiration OCR (Basal), OCR (Maximal), proton leak, ATP production and ECAR (glycolysis and glycolytic capacity), indicating that H/R inhibits oxygen consumption and promoted extracellular acidification (Figure 7C). With JC105 treatment, OCR (Basal and Maximal) was significantly increased along with proton leak and ATP production (Figure 7C). Whereas, ECAR (Glycolytic capacity) was significantly increased, indicating that JC105 ameliorated mitochondrial function and glycolytic capacity of H9c2 cells in H/R (Figure 7F). The ratio of OCR/ECAR was not affected under normoxia (Figure 7G) but significantly reduced in H/R (Figure 7H). But JC105 treatment increased the OCR/ECAR ratio in H/R (Figure 7H). However, U0126 significantly inhibited the OCR/ECAR ration despite in the presence of JC105 in H/R (Figure 7H). Interestingly, ERK inhibitor U0126 significantly inhibited all the protective bioenergetic effects of JC105 in H/R (Figure 7A‐H).

### 3.6 U0126 blocks JC105‐induced cytoprotection

**FIGURE 8:**
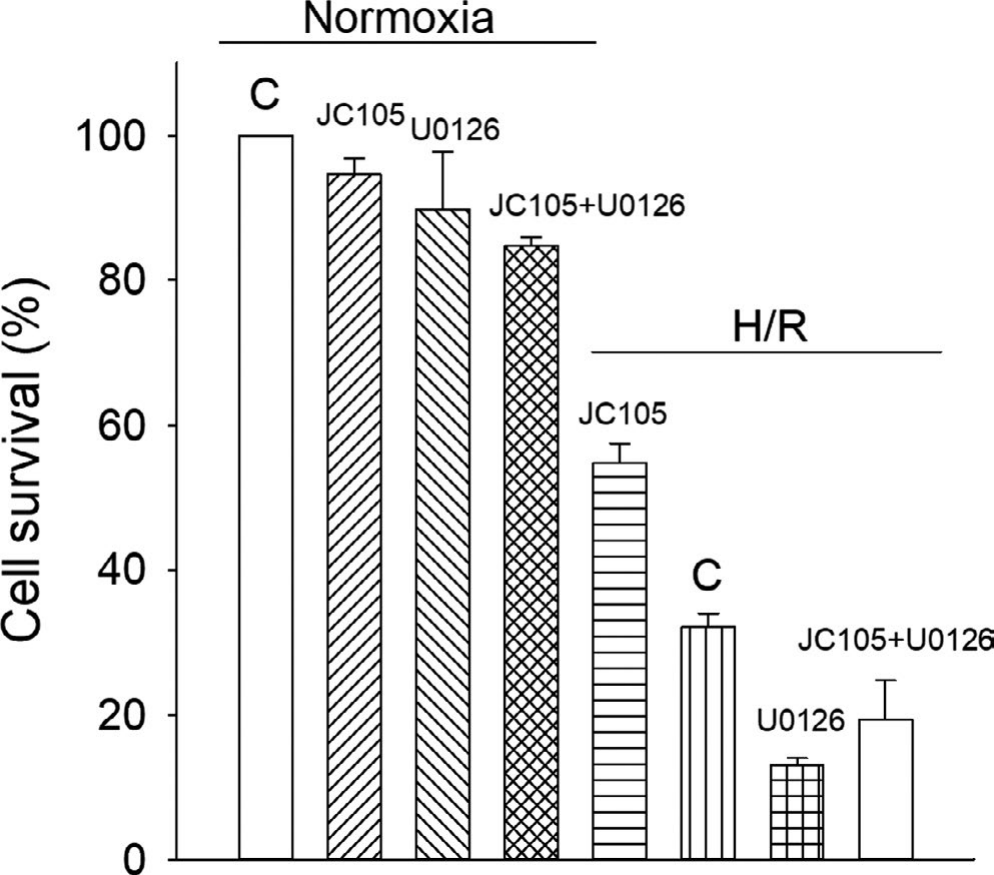
U1026 inhibits JC105‐induced cytoprotection. Bar graph depicting cell survival under normoxic conditions and under hypoxia‐reoxygenation (H/R) under depicted conditions. Each bar represents mean ± SEM (n = 3‐10)

If JC105‐induced phosphorylation of ERK1 and ERK1 mediates cytoprotection under conditions of hypoxia‐reoxygenation, inhibition of this phosphorylation by U0126 should inhibit cytoprotection. Indeed, U0126 (10 μM) abolished cytoprotective effect afforded by JC105 (Figure 8).

## 4 DISCUSSION

JC105 is a novel derivative of isosteviol we recently synthesized. In our initial experiments, we have determined that this compound protects zebrafish embryos against doxorubicin‐induced cardiotoxicity in vivo, which suggested a potential cardioprotective properties. Therefore, we have assessed potential cardioprotection afforded by JC105 using H9c2 cell line. H9c2 cells are well‐established experimental model to study cardioprotection, and this cell line was instrumental in establishing important cardioprotective signalling pathways.^3^, ^4^ JC105 significantly increased survival of H9c2 cells in hypoxia‐reoxygenation suggesting that this compound is cardioprotective under conditions that are clinically relevant.

ERK signalling has been established as a ‘player’ in cardiac hypertrophy development as a ‘good’ one to stimulate or as a ‘bad’ actor to be mitigated, depending on the pathophysiological context.^7^ In addition, it has been suggested that ERK1/2 also mediates cardioprotection.^6^ In the present study, JC105 did not affect ERK1/2 phosphorylation under normoxic conditions, which would imply that this compound has no effect on ERK1/2 phosphorylation. However, JC105 increased phosphorylation of ERK1/2 in cells exposed to hypoxia‐reoxygenation showing that there is a condition‐dependent stimulation of ERK1/2 by JC105. At this moment in time, we are not aware of any compound that would have similar effect on ERK1/2 only under stress conditions. We have previously shown both in vitro and in vivo that small changes in oxygen tensions stimulate ERK1/2 phosphorylation.^5^, ^8^ Activation of ERK1/2 was associated with increase in level of SUR2A.^5^, ^8^ SUR2A is a regulatory subunit of sarcolemmal K_ATP_ channels, which levels regulate myocardial resistance to metabolic stress.^9^, ^10^ If JC105 activates ERK1/2, it was logical to expect that it would also increase SUR2A levels. Indeed, our Western blotting experiments demonstrate that SUR2A followed ERK1/2 pattern, that is JC105 did not affect SUR2A levels in normoxia, but it increased SUR2A levels in hypoxia‐reoxygenation. These findings are another evidence that JC105 activates ERK1/2 specifically during hypoxia‐reoxygenation and it also explains cardioprotection afforded by JC105 as it is well established that increased levels of SUR2A mediate cardioprotection by regulating timing of K_ATP_ channels opening during stress and preventing stress‐induced decrease in subsarcolemmal levels of ATP.^11^, ^12^

However, ERK1/2 activation can have many other effects with potential cardioprotective outcome in addition to SUR2A regulation. As an example, it has been shown that ERK1/2 promotes cell survival by activating pro‐survival BCL2 proteins (BCL‐2, BCL‐XL and MCL‐1) and repressing pro‐death proteins (BAD, BIM, BMF and PUMA). More recently, a link between ERK1/2 signalling, DRP1 and the mitochondrial fission machinery has been established.^6^ We have recently published that isosteviol protects the myocardium against ischaemia‐reperfusion by inhibiting DRP1 phosphorylation and mitochondrial fission.^13^ Here, we have found that JC105 stimulates DRP1 phosphorylation and it has been previously shown that inhibition of DRP1 is cardioprotective.^13^, ^14^ Conversely, it has been suggested that phosphorylation of DRP1 mediates adrenergic signalling activation‐induced alterations in mitochondrial morphology and function that contributes to the development and progression of heart failure.^15^ From this prospective, our results are unexpected. It is quite possible that modest phosphorylation of DRP1 does not induce significant intracellular damage while simultaneous increase in SUR2A mediates cardioprotection. In support of this, notion is our recent finding that α‐adrenergic stimulation induced cardioprotection by increasing K_ATP_ channel levels despite this being a signal phosphorylating DRP1.^16^ As it is known that ERK1/2 phosphorylates DRP1,^17^ phosphorylation of DRP1 further confirms that JC105 indeed activates ERK1/2 signalling pathway.

U0126 is a well‐established inhibitor of ERK1/2.^18^ If phosphorylation of ERK1/2 is not just an epiphenomenon, but genuinely responsible for JC105‐induced cytoprotection, U0126 should inhibit JC105‐induced cytoprotection. Our Western blotting experiments confirmed that U0126 inhibits ERK1/2 phosphorylation during hypoxia‐reoxygenation and that JC105 is unable to stimulate ERK1/2 phosphorylation in the presence of U0126. Phosphorylation of DRP1 followed the same pattern confirming that ERK1/2 targets DRP1 and that blocking ERK1/2 blocks all consequences of ERK1/2 activation. Moreover, our experiments showed that U0126 did not block other cardioprotective kinases such as AKT and AMPK^4^ demonstrating high specificity of U0126. These experiments also showed that JC105 did not activate AKT or AMPK signalling pathway suggesting that these major cardioprotective signalling pathways are not regulated by JC105. Cytoprotection afforded by JC105 was inhibited by U0126, which strongly suggests a causal link between JC105‐induced cytoprotection and ERK1/2 activation.

Our seahorse experiments further demonstrate that JC105 does not interfere with intracellular physiology at rest, while it dramatically improves mitochondrial functions in hypoxia‐reoxygenation. As mitochondria are targets of ERK1/2 and a crucial factor in cardioprotection,^19^ these results provide further evidence in favour of ERK1/2 involvement in JC105‐mediated cardioprotection.

Our findings demonstrate that (a) JC105 protects H9c2 cells against hypoxia‐reoxygenation and that (b) this effect is mediated via ERK1/2. It is particularly interesting that JC105 does not affect ERK1/2 signalling at rest and it induces ERK1/2 phosphorylation only during stress conditions. This unique property makes JC105 a compound that deserves to be further tested as a drug against conditions where cardioprotection is desirable including ischaemia‐reperfusion. It is likely that a selective effect on cardiomyocytes under stress alone makes this compound much safer than it would be if ERK1/2 was activated in healthy cells as well. Taken all together, JC105 seems to be a compound that deserves to be further tested as a potential cardioprotective agent that can be introduced in clinical practice. Clearly, in vivo studies are in order to confirm the cardioprotective properties of JC105. However, it should be pointed out that, so far, any compound that was cardioprotective at in vitro level was also cardioprotective in in vivo experimental models.^4^

In conclusion, we have shown that JC105 harbours cardioprotective properties that are associated with ability of this compound to selectively activate ERK1/2 signalling in cells under stressful conditions.

## CONFLICT OF INTEREST

The authors confirm that no conflict of interest exists.

## AUTHORS' CONTRIBUTIONS

KSMA, JR, N. F. and AJ: Contribution to the acquisition, analysis, interpretation of data and contribution to writing of the manuscript. WT: Initiation, designing and supervision of the study, analysis of the data and drafting the manuscript. All authors: Reading and approving the final version of the paper.

## Supporting information

Fig S1

Fig S2

## Data Availability

Data will be available from the corresponding author (WT) upon reasonable request.
