# Supplementary figures and images for: Cardioprotection by isosteviol derivate JC105: A unique drug property to activate ERK1/2 only when cells are exposed to hypoxia‐reoxygenation
Source: J Cell Mol Med. 2020 Aug 14;24(18):10924–34. doi: 10.1111/jcmm.15721 (PMC7521240; doi:10.1111/jcmm.15721)

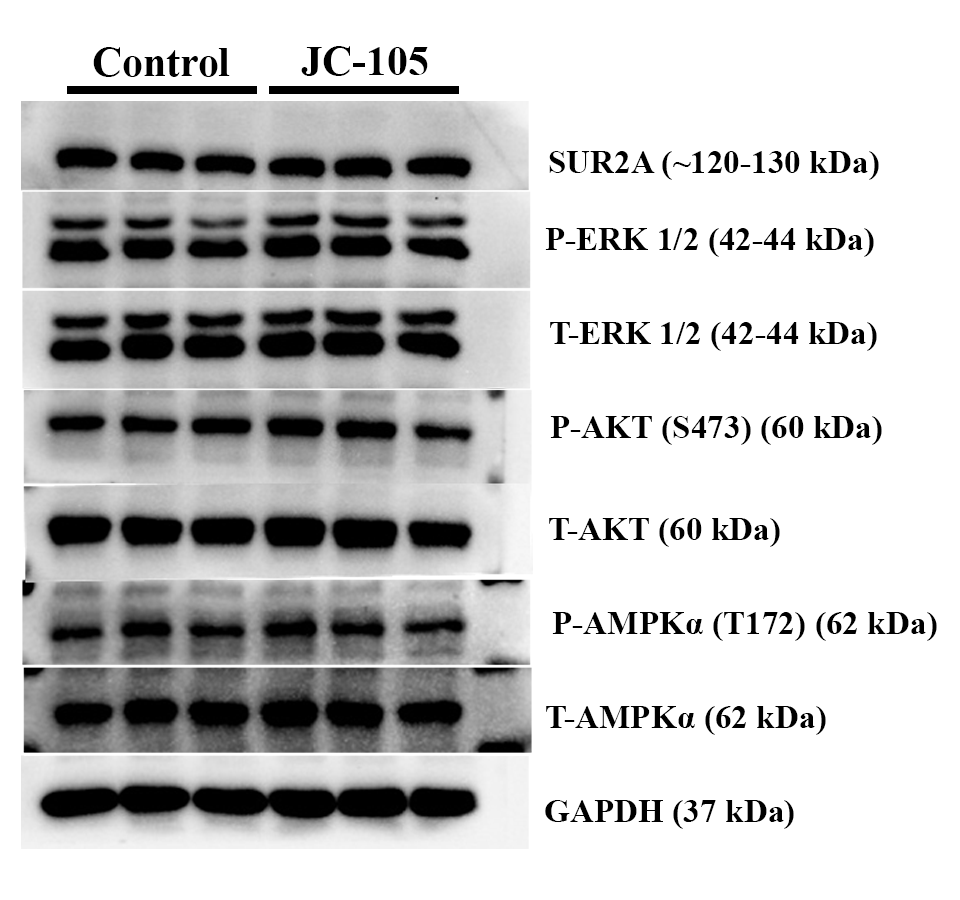

Supplement: Supplementary file 1 — Fig S1 [file JCMM-24-10924-s001.tif]

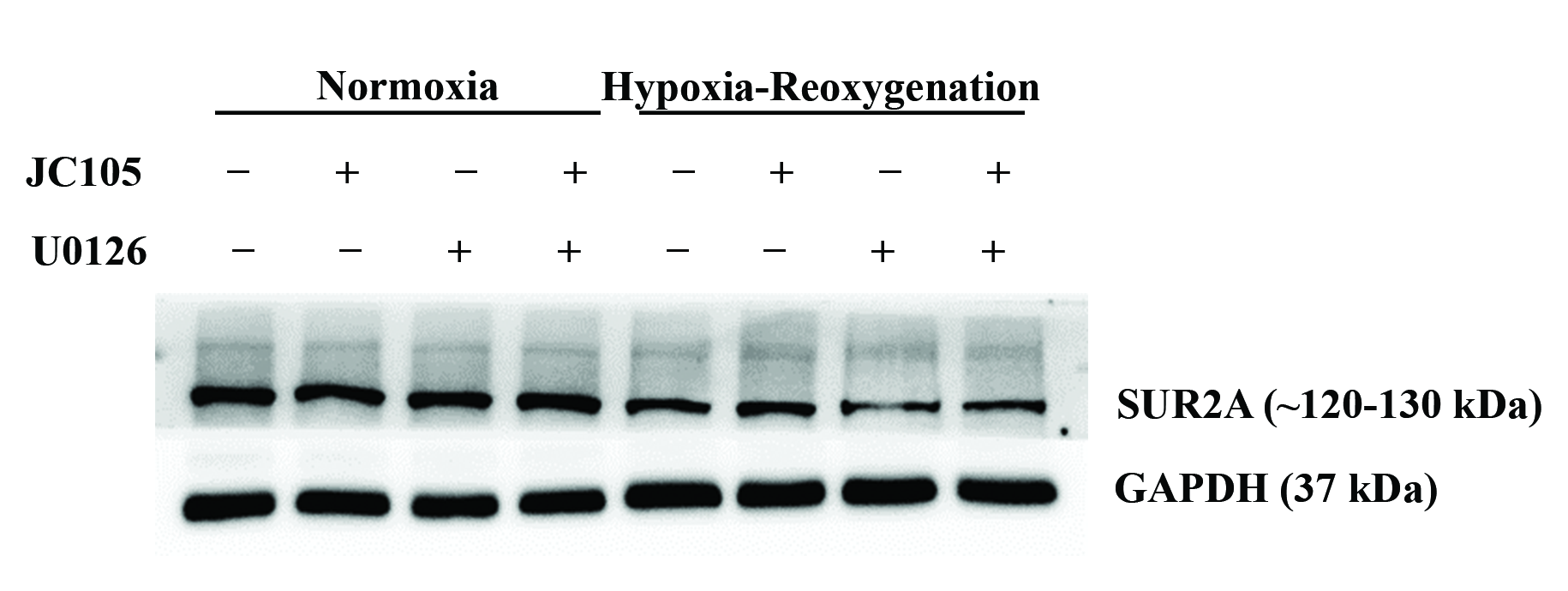

Supplement: Supplementary file 2 — Fig S2 [file JCMM-24-10924-s002.tif]
